# Interaction of Intraprocedural Antiplatelets and Intravenous Thrombolysis in Acute Intracranial Stenting: RESISTANT Registry Subanalysis

**DOI:** 10.1002/acn3.70449

**Published:** 2026-06-09

**Authors:** Aaron Rodriguez‐Calienes, Leonardo Cruz‐Criollo, Eric Kontowicz, Marta Olivé‐Gadea, Francesco Diana, Johannes Kaesmacher, Adnan Mujanovic, Serdar Geyik, Songul Senadim, Amedeo Cervo, Andrea Salcuni, Mariangela Piano, Manuel Moreu, Alfonso López‐Frías, Ameer E. Hassan, Samantha Miller, Elena Zapata‐Arriaza, Asier de Albóniga‐Chindurza, Mauro Bergui, Stefano Molinaro, João André Sousa, Fábio Gomes, João Sargento‐Freitas, Andrea Alexandre, Alessandro Pedicelli, Jeremy Hofmeister, Paolo Machi, Luca Scarcia, Erwah Kalsoum, Jose Amorim, Torcato Meira, Leonardo Renieri, Francesco Capasso, Daniele Romano, Eduardo Bárcena‐Ruiz, David Seoane, Mohamad Abdalkader, Piers Klein, Thanh N. Nguyen, Catarina Perry da Câmara, Anderson Brito, Nashwa Abdelhakim, Isabel Fragata, Dileep R. Yavagal, Jude H. Charles, Jose Rodriguez Castro, Pedro Vega, Atilla Özcan Özdemir, Zehra Uysal Kocabaş, Stanislas Smajda, Sadiq Al Salman, Jane Khalife, Tudor G. Jovin, Francesco Biraschi, Francesca Richetti, Pedro Castro, Luis Albuquerque, Adnan Siddiqui, Vinay Jaikumar, Pedro Navia, Nikolaos Ntoulias, Marios Psychogios, Mariano Velo, Joaquín Zamarro, Gonzalo de Paco, Yazan Ashouri, Mohammad AlMajali, Juan F. Arenillas, Alicia Sierra, Michele Romoli, João Pedro Marto, Shadi Yaghi, Marc Ribo, Alejandro Tomasello, Manuel Requena, Santiago Ortega‐Gutierrez

**Affiliations:** ^1^ Department of Neurology University of Miami & Jackson Memorial Hospitals Miami Florida USA; ^2^ Facultad de Medicina Humana Universidad Científica del Sur Lima Perú; ^3^ Department of Neurology University of Iowa Iowa City Iowa USA; ^4^ Stroke Unit Hospital Universitari Vall d’Hebron Barcelona Spain; ^5^ Interventional Neuroradiology Hospital Universitari Vall d’Hebron Barcelona Spain; ^6^ University Institute of Diagnostic and Interventional Neuroradiology Inselspital, University of Bern Bern Switzerland; ^7^ Department of Radiology, IAU Stroke Center Istanbul Aydın University Istanbul Turkey; ^8^ Department of Neurology, IAU Stroke Center Istanbul Aydın University Istanbul Turkey; ^9^ Department of Neuroradiology ASST Grande Ospedale Metropolitano Niguarda Milan Italy; ^10^ Interventional Neuroradiology Unit, Department of Radiology Hospital Clínico Universitario San Carlos Madrid Spain; ^11^ Department of Neurology Valley Baptist Medical Center Harlingen Texas USA; ^12^ Neurology Department Biomedicine Institute of Seville (IBIS), University Hospital Virgen del Rocio Sevilla Spain; ^13^ Neuroradiology Unit A.O. Città della Salute e della Scienza Turin Italy; ^14^ Department of Neurology Coimbra University Coimbra Portugal; ^15^ UOSA Neuroradiologia Interventistica, Fondazione Policlinico Universitario A. Gemelli IRCCS Rome Italy; ^16^ Diagnostic and Interventional Neuroradiology Geneva University Hospitals Geneva Switzerland; ^17^ Department of Neuroradiology Henri Mondor Hospital Creteil France; ^18^ Neuroradiology Department Hospital de Braga Braga Portugal; ^19^ Interventional Neuroradiology Department Careggi University Hospital Florence Italy; ^20^ Neuroradiology, University Hospital “San Giovanni di Dio e Ruggi d’Aragona” Salerno Italy; ^21^ Department of Radiology Hospital Universitario 12 de Octubre Madrid Spain; ^22^ Department of Neurology Hospital Universitario 12 de Octubre Madrid Spain; ^23^ Department of Radiology, Boston Medical Center Boston University Chobanian and Avedisian School of Medicine Boston Massachusetts USA; ^24^ Department of Neurology, Boston Medical Center Boston University Chobanian and Avedisian School of Medicine Boston Massachusetts USA; ^25^ Department of Neuroradiology Centro Hospitalar Universitário Lisboa Central Lisbon Portugal; ^26^ Department of Radiology Hospital Universitario Central de Asturias Oviedo Spain; ^27^ Department of Neurology Eskisehir Osmangazi University School of Medicine Eskisehir Turkey; ^28^ Department of Interventional Neuroradiology Rothschild Foundation Hospital Paris France; ^29^ Department of Neurology Cooper Neurological Institute Camden New Jersey USA; ^30^ Department of Human Neurosciences, Interventional Neuroradiology, Policlinico Umberto I Sapienza University of Rome Rome Italy; ^31^ Department of Neurology Centro Hospitalar Universitário de São João Faculty of Medicine University of Porto Porto Portugal; ^32^ Department of Neuroradiology Centro Hospitalar Universitário São João Porto Portugal; ^33^ Department of Neurosurgery and Radiology and Canon Stroke and Vascular Research Center University at Buffalo Jacobs School of Medicine and Biomedical Sciences Buffalo New York USA; ^34^ Department of Neuroradiology Hospital Universitario La Paz Madrid Spain; ^35^ Department of Diagnostic and Interventional Neuroradiology University Hospital Basel Basel Switzerland; ^36^ Neuroradiology Unit, Department of Biomedical Sciences and Morphological and Functional Imaging University of Messina Messina Italy; ^37^ Interventional Neuroradiology, Radiology, Hospital Clínico Universitario Virgen de la Arrixaca Murcia Spain; ^38^ Neuroscience and Stroke Program, Bon Secours Mercy Health St Vincent Hospital Toledo Ohio USA; ^39^ Stroke Program, Department of Neurology Hospital Clínico Universitario Valladolid Spain; ^40^ Neurology and Stroke Unit, Department of Neuroscience Bufalini Hospital Cesena Italy; ^41^ Department of Neurology Hospital de Egas Moniz, Centro Hospitalar Lisboa Ocidental Lisbon Portugal; ^42^ Department of Neurology The Warren Alpert Medical School of Brown University Providence Rhode Island USA

**Keywords:** antiplatelets, intracranial stenting, thrombolysis

## Abstract

**Introduction/Objective:**

Acute intracranial stenting during endovascular thrombectomy (EVT) for ischemic stroke requires intraprocedural antiplatelet therapy (APT) to maintain patency. However, the hemorrhagic risk of combining APT with intravenous thrombolysis (IVT) remains uncertain. We evaluated the safety of IVT combined with conservative versus aggressive intraprocedural APT in patients requiring stenting during EVT.

**Methods:**

This multicenter RESISTANT registry subanalysis (2016–2023) included 823 adults. APT was categorized as conservative (aspirin +/− oral P2Y12) or aggressive (including GPIIb/IIIa inhibitors or cangrelor). The primary outcome was a composite of symptomatic intracranial hemorrhage (sICH) and parenchymal hematoma (PH1/PH2). Multivariable logistic regression assessed associations and interactions between IVT and APT.

**Results:**

A total of 823 patients were included: 44 (5.3%) received IVT + conservative APT, 130 (15.8%) No IVT + conservative APT, 145 (17.6%) IVT + aggressive APT, and 504 (61.2%) No IVT + aggressive APT. Frequencies of sICH‐PH1‐PH2 were 9.3% with IVT + conservative APT, 10.7% with IVT + aggressive APT, 3.2% with No IVT + conservative APT, and 9.9% with No IVT + aggressive APT. In multivariable analysis without interaction terms, neither IVT (aOR 1.18, 95% CI 0.58–2.27; *p* = 0.64) nor aggressive APT (aOR 2.10, 95% CI 0.92–5.69; *p* = 0.10) was independently associated with increased risk of sICH‐PH1‐PH2. However, in the interaction model, IVT within the conservative‐APT stratum (aOR 5.84, 95% CI 1.07–43.92; *p* = 0.05) and aggressive APT within the no‐IVT stratum (aOR 4.81, 95% CI 1.41–30.22; *p* = 0.03) were each associated with higher odds of sICH‐PH1‐PH2, while the IVT‐by‐APT interaction term was < 1 (aOR 0.15, 95% CI 0.02–0.94; *p* = 0.05), indicating attenuation of the joint effect on the multiplicative odds scale.

**Conclusion:**

Among patients requiring intracranial stenting during EVT, we found no evidence that IVT and aggressive intraprocedural APT act synergistically to increase hemorrhagic risk. Rather, the negative IVT‐by‐APT interaction suggested attenuation of the joint effect on the multiplicative odds scale, although patients receiving both therapies remained at increased hemorrhagic risk relative to the reference group.

## 1 Introduction

Endovascular thrombectomy (EVT) has limitations, as up to 20% of acute ischemic stroke (AIS) patients with large vessel occlusions (LVOs) do not achieve successful reperfusion [1, 2, 3]. Causes of EVT failure are varied, but intracranial atherosclerotic disease (ICAS) is among the most common when LVO is present (ICAS‐LVO) [4, 5, 6]. In such cases, acute intracranial stenting has been adopted, either as a rescue strategy after failed thrombectomy or as first‐line treatment with upfront stent deployment before thrombectomy attempts [7].

Intraprocedural antiplatelet therapy (APT) is crucial during acute intracranial stenting in EVT to prevent acute in‐stent thrombosis and subsequent restenosis or reocclusion [8]. Currently, there is no consensus on the optimal intraprocedural APT regimen [3], resulting in real‐world decisions that are guided by institutional protocols. A primary therapeutic objective is to achieve immediate antiplatelet effect to secure stent patency, while carefully balancing the risk of intracranial hemorrhage (ICH). This trade‐off is especially critical when potent, short‐acting regimens (e.g., intravenous glycoprotein IIb/IIIa inhibitors or cangrelor) are co‐administered with IVT using alteplase or tenecteplase [9, 10, 11].

The interaction between various APT regimens and thrombolytic therapy is still poorly understood due to limited evidence on safety outcomes and the absence of standardized treatment protocols [3]. Variability in APT regimens, combined with insufficient data on their interaction with IVT, leaves the optimal antithrombotic strategy for intracranial stenting uncertain [11]. We aimed to assess the hemorrhagic risk across commonly used intraprocedural APT strategies (conservative vs. aggressive) and to test for effect modification by prior IVT in patients undergoing acute intracranial stenting during EVT.

## 2 Methods

### 2.1 Study Design

This study was a subanalysis of data from the RESISTANT registry, which was an international, multicenter, retrospective patient‐level registry designed to evaluate the real‐world use of intracranial stenting during EVT in patients with AIS. Details of RESISTANT were published previously. Briefly, from January 2016 to June 2023, consecutive patients aged > 18 years with AIS who underwent intracranial stenting as part of EVT were enrolled across 36 comprehensive stroke centers spanning seven countries and three continents. Patients who underwent intracranial angioplasty without subsequent stenting were excluded. Although RESISTANT was a retrospective registry, all data were prospectively collected at each participating center using standardized data collection protocols. Patient information was de‐identified locally within each center's registry. Institutional review board (IRB) approval was obtained at all participating institutions. This study adheres to the Strengthening the Reporting of Observational Studies in Epidemiology (STROBE) guidelines [12].

### 2.2 Study Population and Interventions

Indications for intracranial stenting during EVT were determined by time windows, imaging findings, comorbidities, and established guidelines [3, 13]. Stenting was performed according to patient condition and local protocols. When not contraindicated, IVT was administered per international guidelines [3, 13]. The choice of endovascular approach was left to the operator, and stenting strategies were not standardized across centers. Stenting was performed with or without balloon angioplasty, either after failed thrombectomy (rescue stenting) or as a first‐line strategy without prior thrombectomy passes.

Intraprocedural APT was categorized a priori as conservative versus aggressive. A conservative strategy consisted of intravenous or oral aspirin alone, or aspirin plus an oral P2Y12 inhibitor (clopidogrel or ticagrelor). Aggressive APT was defined as any regimen including an intravenous GP IIb/IIIa inhibitor or intravenous cangrelor. GP IIb/IIIa inhibitors (abciximab, tirofiban, eptifibatide) were administered intravenously using standard regimens. IVT (alteplase or tenecteplase) followed guideline‐based indications. For the purpose of this study, four exposure groups were formed: conservative APT without IVT, aggressive APT without IVT, conservative APT with IVT, and aggressive APT with IVT.

Regardless of intraprocedural APT, dual APT was often introduced after the procedure on a case‐by‐case basis, guided by imaging findings (infarct size, ICH, stent patency) and the patient's neurological status.

### 2.3 Data Collection

Baseline data collected included age, sex, pre‐stroke modified Rankin Scale (mRS) score, prior history of stroke, smoking status, other vascular risk factors, and a previous diagnosis of ICAS. Stroke severity was assessed using the National Institutes of Health Stroke Scale (NIHSS) at admission, 24 h, and either at 5 days or prior to discharge. Workflow times, including symptom onset, hospital arrival, treatment initiation, and time to reperfusion, were documented. Imaging data included baseline Alberta Stroke Program Early Computed Tomography Score (ASPECTS), occlusion location, perfusion imaging findings (when available), and follow‐up computed tomography (CT) for hemorrhage detection. Procedural variables recorded by local investigators included vascular access site, number of thrombectomy passes prior to stent deployment, use of pre‐stenting angioplasty, number and type of stents deployed, intraprocedural antiplatelet therapy, and degree of reperfusion before and after stenting based on the mTICI scale [14]. Intraprocedural complications, such as subarachnoid hemorrhage, vessel perforation, or dissection, were also noted. Stent patency at 24 h was assessed using either CT angiography or transcranial ultrasound.

### 2.4 Study Outcomes

The primary outcome was a composite of symptomatic intracranial hemorrhage (sICH), parenchymal hematoma type 1 (PH1), and parenchymal hematoma type 2 (PH2), adjudicated using the Heidelberg Bleeding Classification [15], designed to improve sensitivity and capture the relevant elements potentially associated with clinical outcomes. Each component of the composite outcome was also analyzed separately. sICH was defined as any intracranial hemorrhage with new or worsening neurological deficits within 24 h of EVT.

Secondary outcomes were any ICH, successful reperfusion (mTICI 2b‐3), intraprocedural stent occlusion, 90‐day favorable outcome (mRS 0–2), and 90‐day mortality.

### 2.5 Statistical Analysis

Descriptive statistics were used to summarize categorical and continuous variables. Continuous variables are reported as medians and interquartile ranges (IQR) based on their distribution. Counts and percentages were used to summarize categorical variables. Shapiro–Wilk test and visual inspection of histograms were employed to assess the normality of continuous variable distributions. For univariate analyses, chi‐square or Fisher's exact tests were used for categorical variables, while Kruskal–Wallis tests were applied to continuous variables to detect significant differences across the combinations of APT regimen and IVT administration.

Two multivariable logistic regression models were constructed for our primary analysis. The main‐effects model adjusted for IVT and APT regimen separately, along with clinically relevant covariates selected a priori: age, admission NIHSS score, onset‐to‐recanalization time, final TICI grade, ASPECTS, and the number of passes before rescue stenting. The second model assessed the interaction between IVT and APT regimens, adjusting for the same covariates as above with the addition of an IVT × APT interaction term. All modeling was restricted to patients with complete outcome and covariate data. All covariates were modeled as fixed effects. Random effects for study site were not included, as mixed‐effect models yielded results comparable to fixed‐effect models. Odds ratios (ORs) and 95% confidence intervals (CIs) were reported for all variables, except for the interaction term, where only the *p*‐value was reported. Given the rarity of the outcomes (< 10% in our total population), odds ratios from logistic regression models were assumed to approximate risk ratios. An interaction plot was generated to explore and visualize the interaction effect between IVT administration and APT regimen on the primary outcome across different APT levels.

To illustrate and contextualize the interaction between APT category and IVT, we performed a stratified multivariable analysis. Data were stratified by IVT and, within each stratum, a logistic regression model was fitted adjusting for the same covariates as the primary analysis, excluding IVT status and the APT–IVT interaction term.

To further explore the interaction between aggressive APT and IVT, we performed a sub‐group analysis restricted to individuals who received both IVT and aggressive APT treatments. In this sub‐group analysis, we fit separate unadjusted logistic models assessing the associations of selected clinical covariates with the composite primary outcome of sICH‐PH1‐PH2. Covariates included age (dichotomized at 70 years), number of passes (dichotomized at ≥ 3), ASPECTS (> 8), receipt of any GPI versus cangrelor, receipt of heparin, sex, NIHSS (> 14), and IV APT alone versus IV APT with oral APT.

To evaluate the association between APT regimen, IVT receipt, and our secondary outcomes, we fit separate multivariable logistic regression models for each outcome. Models for sICH and any ICH were adjusted for age, NIHSS, onset‐to‐recanalization time, ASPECTS, reperfusion status, and number of passes. Models for 90‐day mortality and favorable functional outcome were adjusted for age, NIHSS, onset‐to‐recanalization time, and ASPECTS. Lastly, models for successful reperfusion and intraprocedural stent occlusion were adjusted for onset‐to‐recanalization time and the presence of a tandem lesion. In all cases, APT regimen and IVT receipt were included as independent variables without an interaction term. All statistical tests were considered significant at a two‐sided alpha level of ≤ 0.05. All analysis was performed in R version 4.3.1 (R Foundation for Statistical Computing, Vienna, Austria).

## 3 Results

### 3.1 Patient Population

The flowchart of the patient selection process is illustrated in Figure S1. A total of 823 patients from the RESISTANT registry were included, 130 (15.8%) were treated with conservative APT without IVT, 504 (61.2%) with aggressive APT without IVT, 44 (5.3%) with conservative APT with IVT, and 145 (17.6%) with aggressive APT with IVT. Table S1 provides a detailed breakdown of the intraprocedural antiplatelet regimens used.

Table 1 shows the baseline characteristics for each therapeutic approach used. Median age, sex distribution, and most comorbidities were similar across treatment groups. Prior stroke (38.3% in No IVT + conservative APT vs. 16.1% in IVT + aggressive APT; *p* < 0.001), prior antiplatelet use (41.4% vs. 26.1%; *p* = 0.007), and known ICAS (15.5% vs. 2.8%; *p* = 0.001) were more frequent in the No IVT + conservative APT group compared with IVT + aggressive APT. Hypertension was also significantly different in the No IVT groups (76.7% and 75.6%) than in IVT groups (69.8% and 63.2%; *p* = 0.019). Tandem occlusions were more often in No IVT + conservative APT (27%) and IVT + conservative APT (33.3%) than in IVT + aggressive APT (8.3%; *p* < 0.001). Time from symptom onset to recanalization was shortest in IVT + aggressive APT (313 min [IQR: 238–429]) and longest in No IVT + conservative APT (455 min [IQR 256–816]; *p* < 0.001). First‐line stenting was most frequently used in No IVT + conservative APT (20% vs. 6.8% in IVT + conservative APT; *p* = 0.022).

**TABLE 1:** Baseline characteristics of the study participants by intravenous thrombolysis (IVT) use and intraprocedural antiplatelet (APT) regimen.

	Total (*N* = 823)	No IVT		IVT		*p*
		Conservative APT (*N* = 130)	Aggressive APT (*N* = 504)	Conservative APT (*N* = 44)	Aggressive APT (*N* = 145)	
Age, year, median [IQR]	67 [59–77]	66 [58.2–76.8]	68 [59–77]	64 [55–75.7]	67 [56–77]	0.409
Female, *n* (%)	529 (64.4)	91 (70)	316 (62.8)	30 (68.2)	92 (63.4)	0.448
Comorbidities, *n* (%)						
Hypertension	598 (73.3)	99 (76.7)	378 (75.6)	30 (69.8)	91 (63.2)	**0.019**
Hyperlipidemia	371 (45.5)	66 (51.6)	231 (46.2)	19 (44.2)	55 (38.2)	0.163
Coronary artery disease	106 (13)	23 (18)	67 (13.4)	4 (9.3)	12 (8.4)	0.109
Diabetes mellitus	290 (35.6)	46 (35.9)	189 (37.8)	14 (32.6)	41 (28.7)	0.238
Atrial fibrillation	117 (14.4)	18 (14.1)	80 (16)	5 (11.6)	14 (9.8)	0.285
Prior stroke	220 (27)	49 (38.3)	142 (28.4)	6 (14)	23 (16.1)	**< 0.001**
Smoking	336 (41.7)	59 (46.5)	205 (41.5)	14 (32.6)	58 (41.1)	0.443
Prior antiplatelet, *n* (%)	236 (29.1)	53 (41.4)	137 (27.5)	9 (20.9)	37 (26.1)	**0.007**
Prior oral anticoagulant, *n* (%)	126 (15.5)	18 (14.1)	90 (18)	1 (2.3)	17 (12)	**0.021**
Known ICAS, *n* (%)	60 (7.4)	20 (15.5)	34 (6.8)	2 (4.7)	4 (2.8)	**0.001**
Baseline NIHSS, median [IQR]	13 [8–19]	11.5 [6–19]	12 [8–19]	15 [9–21]	15 [8–19]	0.176
ASPECTS, median [IQR]	9 [8–10]	9 [8–10]	9 [8–10]	8 [7.5–9.5]	9 [8–10]	0.147
Site of occlusion, *n* (%)						0.845
ICA‐T	141 (17.2)	24 (18.5)	91 (18.1)	5 (11.4)	21 (14.5)	
MCA‐M1	377 (45.9)	58 (44.6)	224 (44.5)	25 (56.8)	70 (48.3)	
MCA‐M2	60 (7.3)	5 (3.8)	37 (7.4)	5 (11.4)	13 (9.0)	
MCA‐M3/M4	3 (0.4)	0 (0.0)	3 (0.6)	0 (0.0)	0 (0.0)	
ACA‐A1/A2	9 (1.1)	0 (0.0)	6 (1.2)	1 (2.3)	2 (1.4)	
ACA‐A3/A4	1 (0.1)	0 (0.0)	1 (0.2)	0 (0.0)	0 (0.0)	
VA‐V4	61 (7.4)	10 (7.7)	41 (8.2)	2 (4.5)	8 (5.5)	
BA‐proximal	97 (11.8)	19 (14.6)	57 (11.3)	4 (9.1)	17 (11.7)	
BA‐middle	42 (5.1)	9 (6.9)	22 (4.4)	1 (2.3)	10 (6.9)	
BA‐distal	11 (1.3)	3 (2.3)	6 (1.2)	0 (0.0)	2 (1.4)	
PCA‐P1	18 (2.2)	1 (0.8)	14 (2.8)	1 (2.3)	2 (1.4)	
PCA‐P2	2 (0.2)	1 (0.8)	1 (0.2)	0 (0.0)	0 (0.0)	
Tandem occlusions,^a^ *n* (%)	93 (11.4)	34 (27)	33 (6.5)	14 (33.3)	12 (8.3)	**< 0.001**
Onset to recanalization, median [IQR]	388 [252–643]	455 [255.8–815.5]	408 [263–700]	396 [257–520]	313 [238–429]	**< 0.001**
General anesthesia, *n* (%)	422 (51.3)	76 (58.5)	243 (48.2)	23 (52.3)	80 (55.2)	0.067
Stenting approach, *n* (%)						**0.022**
Rescue stenting	721 (87.7)	104 (80)	445 (88.5)	41 (93.2)	131 (90.3)	
First‐line stenting	101 (12.3)	26 (20)	58 (11.5)	3 (6.8)	14 (9.7)	
Number of passes, median [IQR]	2 [1–3]	2 [1–3]	2 [1–3]	2 [1–3]	2 [1–3]	0.318
Thrombectomy technique, *n* (%)						0.547
Stent retriever alone	119 (17)	18 (18.6)	74 (17)	8 (20)	19 (14.7)	
Distal aspiration	158 (22.5)	16 (16.5)	97 (22.2)	7 (17)	38 (29.5)	
Combined	424 (60.4)	63 (64.9)	264 (60.6)	25 (62)	72 (55.8)	
Pre‐stent ballon angioplasty, *n* (%)	420 (51.1)	66 (51.2)	258 (51.2)	22 (50)	74 (51)	0.999
Stent type, *n* (%)						0.623
SES	160 (19.5)	29 (22.5)	96 (19)	10 (23.8)	25 (17.2)	
BMS	660 (80.5)	100 (77.5)	408 (81)	32 (76.2)	120 (82.8)	

*Note:* Bold values indicates statistically significant results with a *p*‐value < 0.05.

Abbreviations: ACA, anterior cerebral artery; ASPECTS, Alberta Stroke Program Early Computed Tomography Score; BA, basilar artery; BMS, balloon‐mounted stent; ICAS, intracranial atherosclerosis stenosis; ICA‐T, terminus portion of the internal carotid artery; IQR, interquartile range; MCA, middle cerebral artery; mRS, modified Rankin Scale; NIHSS, National Institute of Health Stroke Scale; PCA, posterior cerebral artery; SES, self‐expandable stent; VA, vertebral artery.

^a^ Defined as concomitant cervical internal carotid artery severe stenosis or occlusion and an intracranial large‐vessel occlusion or high‐grade intracranial stenosis within the same vascular territory.

### 3.2 Primary Outcome

**FIGURE 1:**
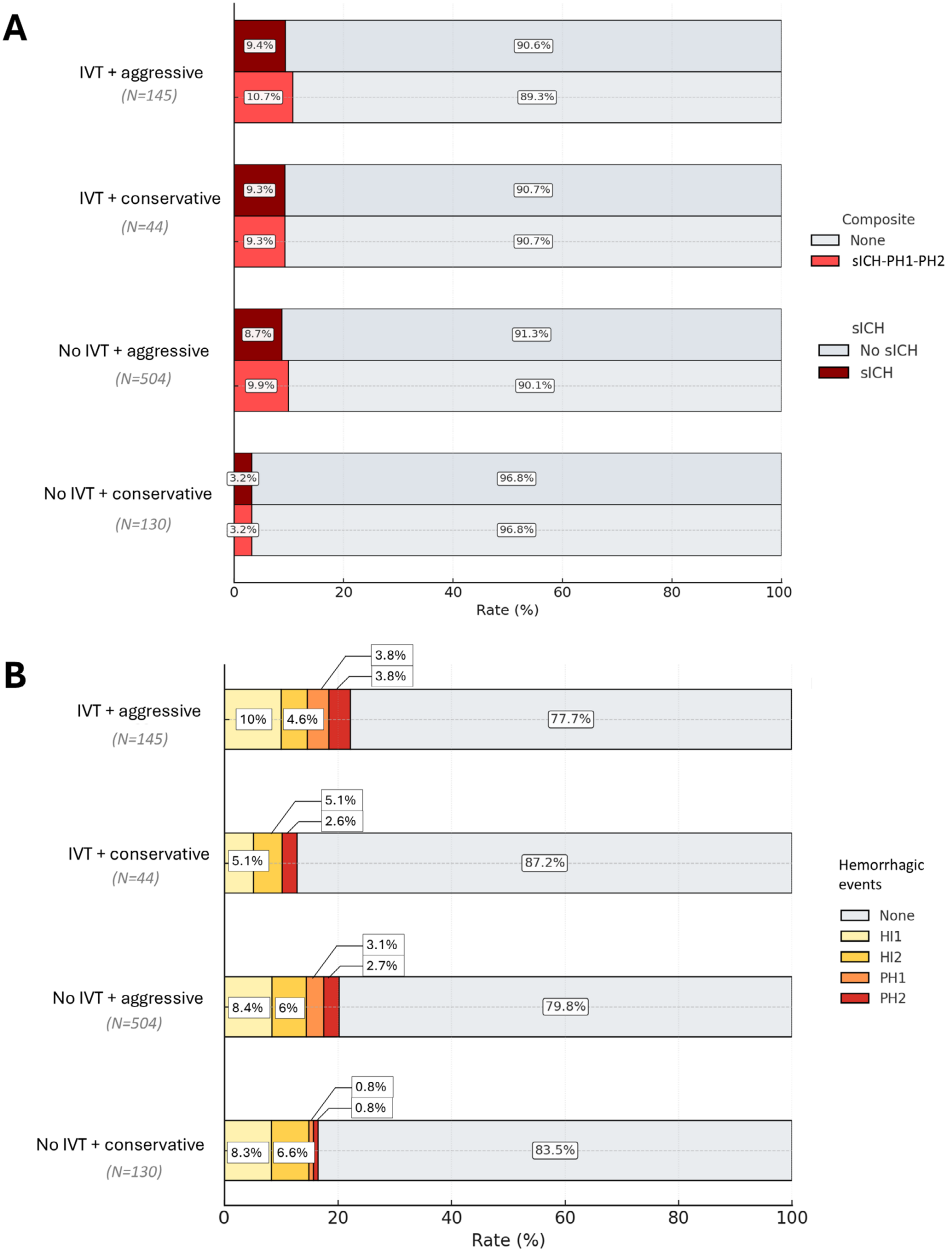
Distribution of hemorrhagic transformation subtypes. Panel (A) shows the composite outcome of sICH‐PH1‐PH2 and sICH, while Panel (B) displays the distribution of hemorrhagic transformation subtypes according to the Heidelberg classification across combinations of intravenous thrombolysis (IVT) use and intraprocedural antiplatelet regimens. PH1, parenchymal hematoma type 1; PH2, parenchymal hematoma type 2; SICH, symptomatic intracranial hemorrhage.

The distribution of the hemorrhagic outcomes across the intervention groups is presented in Figure 1. In univariable analysis, patients in the IVT + aggressive APT group had a higher proportion of the composite sICH‐PH1‐PH2 (10.7%) compared with 3.2% in the No IVT + conservative APT group, although this difference did not reach statistical significance (*p* = 0.062).

In the multivariable main‐effects model without interaction, neither IVT administration (aOR 1.18, 95% CI 0.58–2.27; *p* = 0.64) compared to no IVT, nor aggressive APT (aOR 2.1, 95% CI 0.92–5.69; *p* = 0.10) compared to conservative APT, was independently associated with an increased risk of sICH‐PH1‐PH2 (Table S2).

**FIGURE 2:**
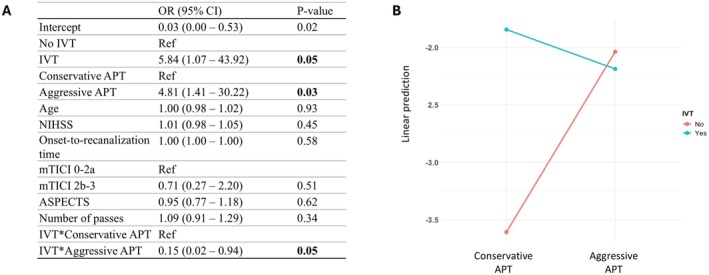
Interaction analysis of intravenous thrombolysis (IVT) with intraprocedural antiplatelet therapy (APT). Panel (A) presents the interaction model for the composite outcome of symptomatic intracranial hemorrhage (sICH), parenchymal hematoma type 1 (PH1), and parenchymal hematoma type 2 (PH2). Panel (B) depicts the corresponding interaction plot, where the crossing of the lines suggests a potential interaction effect. ASPECTS, Alberta Stroke Program Early CT Score; mTICI, modified thrombolysis in cerebral infarction; NIHSS, National Institutes of Health Stroke Scale.

To evaluate potential effect modification between IVT and aggressive APT, an interaction term was introduced into the multivariable model. In this model, IVT within the conservative‐APT stratum (aOR 5.84, 95% CI 1.07–43.92; *p* = 0.05) and aggressive APT within the no‐IVT stratum (aOR 4.81, 95% CI 1.41–30.22; *p* = 0.03) were associated with higher odds of sICH‐PH1‐PH2. The IVT × APT interaction term was < 1 (aOR 0.15, 95% CI 0.02–0.94; *p* = 0.05), indicating attenuation of the joint effect on the multiplicative odds scale rather than synergistic escalation (Figure 2A). Thus, compared with the reference group (no IVT + conservative APT), patients receiving both IVT and aggressive APT remained at elevated odds of sICH‐PH1‐PH2, but the combined association was smaller than expected under a multiplicative model.

Figure S2 illustrates the direction and magnitude of the association between APT category and outcome within each IVT stratum, contextualizing the significant interaction identified in the primary analysis.

### 3.3 Sensitivity Analysis

In the sensitivity analysis restricted to patients who received IVT in combination with aggressive APT (*n* = 145), age, number of passes, ASPECTS, type of aggressive APT, periprocedural heparin use, and NIHSS were not associated with an increased frequency of sICH‐PH1‐PH2 (Figure S3).

**FIGURE 3:**
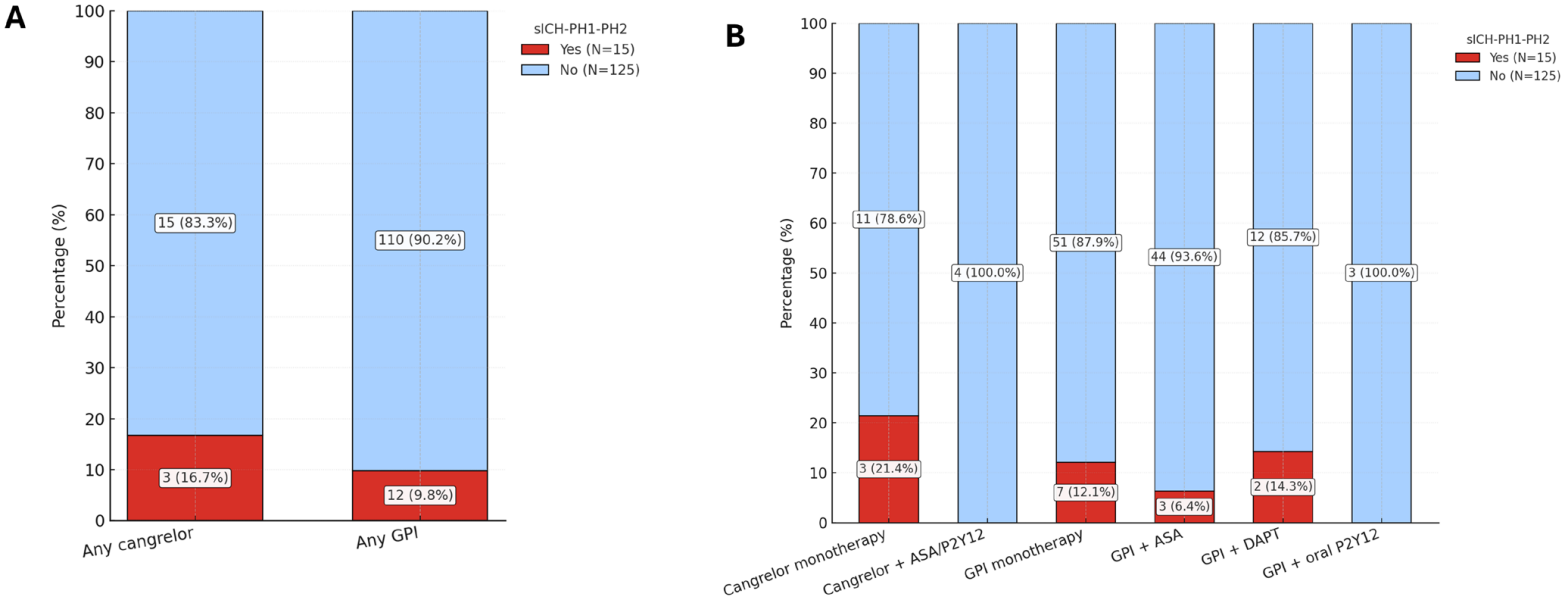
Composite hemorrhagic outcome sICH‐PH1‐PH2 by specific intravenous antiplatelet regimens among patients treated with intravenous thrombolysis. Panel (A) depicts outcomes for any glycoprotein IIb/IIIa inhibitor‐based regimen and any cangrelor‐based regimen, while Panel (B) details outcomes for the specific combinations of each regimen. ASA, aspirin; DAPT, dual antiplatelet therapy; GPI, glycoprotein IIb/IIIa inhibitor.

To further characterize this interaction, we examined sICH‐PH1‐PH2 across aggressive APT regimens in IVT‐treated patients. Crude sICH‐PH1‐PH2 was numerically higher with cangrelor‐based regimens than GPI‐based regimens (16.7% vs. 9.8%) (Figure 3A). Within specific strategies using aggressive ATP in combination with single or dual oral ATP, the highest frequency was observed with cangrelor monotherapy, affecting 21.4% (3/14) of patients (Figure 3B). These exploratory comparisons were not adjusted and are hypothesis‐generating.

### 3.4 Secondary Outcomes

sICH was more frequent in the IVT + aggressive APT group (9.4%) than in the No IVT + conservative APT group (3.2%), while the No IVT + aggressive APT (8.7%) and IVT + conservative APT (9.3%) groups showed similar rates to each other (*p* = 0.145). Any ICH occurred in 17.6% of patients, without significant differences across groups (*p* = 0.511). Intraprocedural stent occlusion was significantly higher in the No IVT + conservative (27%) and IVT + conservative (33.3%) groups compared with the No IVT + aggressive (6.5%) and IVT + aggressive (8.3%) groups (*p* < 0.001). Successful reperfusion was most frequent in the No IVT + aggressive group (92.6%), followed by IVT + aggressive (89%), IVT + conservative (86%), and lowest in No IVT + conservative (82.5%) (*p* = 0.005). At 90 days, favorable outcome (40.8% to 47.6%, *p* = 0.889) and mortality (21.7% to 31.2%, *p* = 0.336) were similar among groups (Table 2).

**TABLE 2:** Outcomes of the study participants by intravenous thrombolysis (IVT) use and intraprocedural antiplatelet (APT) regimen.

	Total (*N* = 823)	No IVT		IVT		*p*
		Conservative APT (*N* = 130)	Aggressive APT (*N* = 504)	Conservative APT (*N* = 44)	Aggressive APT (*N* = 145)	
**Outcomes**						
Any ICH, *n* (%)	145 (17.6)	20 (15.3)	91 (18.1)	5 (11.4)	29 (20)	0.511
HI1^a^	63 (43.4)	10 (50)	38 (41.8)	2 (40)	13 (44.8)	0.763
HI2^a^	43 (29.7)	8 (40)	27 (29.7)	2 (4)	6 (20.7)	
PH1^a^	20 (13.8)	1 (5)	14 (15.4)	0 (0)	5 (17.2)	
PH2^a^	19 (13.1)	1 (5)	12 (13.2)	1 (20)	5 (17.2)	
sICH, *n* (%)	64 (8)	4 (3.2)	43 (8.7)	4 (9.3)	13 (9.4)	0.145
sICH‐PH1‐PH2, *n* (%)	72 (9)	4 (3.2)	49 (9.9)	4 (9.3)	15 (10.7)	0.062
Intraprocedural stent occlusion, *n* (%)	93 (11.4)	34 (27)	33 (6.5)	14 (33.3)	12 (8.3)	**< 0.001**
Successful reperfusion, *n* (%)	736 (90.1)	104 (82.5)	466 (92.6)	37 (86)	129 (89)	**0.005**
90‐day favorable outcome, *n* (%)	326 (42)	51 (40.8)	197 (41.7)	20 (47.6)	58 (42)	0.889
90‐day mortality, *n* (%)	211 (27.2)	39 (31.2)	132 (28)	10 (23.8)	30 (21.7)	0.336

*Note:* Bold values indicates statistically significant results with a *p*‐value < 0.05.

Abbreviations: APT, antiplatelet therapy; HI1, petechial hemorrhage type 1; HI2, petechial hemorrhage type 2; ICH, intracranial hemorrhage; IVT, intravenous thrombolysis; PH1, parenchymal hematoma type 1; PH2, parenchymal hematoma type 2; sICH, symptomatic intracranial hemorrhage.

^a^ Each hemorrhage category is defined according to the Heidelberg Classification, with proportions calculated using only patients who developed any ICH as the denominator.

In adjusted models, IVT was not associated with sICH, any ICH, successful reperfusion, intraprocedural stent occlusion, or 90‐day outcomes. Aggressive APT was not associated with sICH, any ICH, mortality, or favorable outcome, but was linked to higher odds of successful reperfusion (aOR 2.24, 95% CI 1.18–4.14; *p =* 0.01) and lower odds of intraprocedural stent occlusion (aOR 0.18, 95% CI 0.11–0.30; *p* < 0.01) (Table S3).

## 4 Discussion

We conducted a subanalysis of a large, collaborative international registry encompassing 36 comprehensive stroke centers across 7 countries to evaluate the combined effect of IVT administration and APT on the risk of hemorrhagic complications in patients who underwent intracranial stenting during EVT. In adjusted interaction modeling, IVT within the conservative‐APT stratum and aggressive APT within the no‐IVT stratum were each associated with higher odds of sICH‐PH1‐PH2, whereas the negative IVT × APT interaction indicated attenuation of the joint effect rather than synergistic risk amplification [10].

### 4.1 Safety of Intraprocedural APT With Acute Intracranial Stenting

Only a limited number of studies have evaluated APT regimens in the context of acute intracranial stenting during EVT, and, consistent with our findings, most have centered on GPIIb/IIIa inhibitors [9, 16, 17]. Nonetheless, there is marked heterogeneity in practice, not only in the choice of antiplatelet agent but also in how therapy is initiated (e.g., loading strategies) and in dosing regimens. This variability reflects ongoing uncertainty regarding the optimal approach [9]. In our cohort, the predominant intraprocedural APT regimen was GPIIb/IIIa inhibitor‐based therapy, reflecting operator familiarity and a supportive neurointerventional evidence base (Table S1). Aligning with prior reports, we found that aggressive APT alone compared to conservative APT alone was not associated with an increased risk of sICH‐PH1‐PH2 (Table S2). Notably, intraprocedural stent occlusion occurred more frequently in patients managed without aggressive APT (~30%) than in those receiving aggressive APT (~8%), suggesting a potential protective effect of intensified APT on acute stent patency, although this outcome warrants further dedicated investigation.

Anastasiou et al. observed no significant differences in the frequencies of sICH, any subarachnoid hemorrhage, or 90‐day mortality when comparing intraprocedural GPIIb/IIIa inhibitor use with non‐GPIIb/IIIa regimens in a rescue stenting cohort [9]. Similarly, Baek et al. further that intraprocedural GPIIb/IIIa infusion in rescue stenting was not associated with increased sICH compared with either rescue stenting alone or GPIIb/IIIa infusion alone [16]. Noh et al. found no difference in sICH risk between combined use of GPIIb/IIIa inhibitors with intracranial stenting versus GPIIb/IIIa inhibitor alone or no inhibitor at all [17].

### 4.2 Safety of Prior IVT Before Acute Intracranial Stenting

In our multivariable analysis, prior IVT before intracranial stenting was not associated with increased risk sICH‐PH1‐PH2 (Table S2). This observation is consistent with prior studies, which have similarly found no excess hemorrhage risk associated with IVT in this setting. Stracke et al. reported no increased hemorrhage risk in patients who had received IVT prior to emergent intracranial stenting [18]. Mohammaden et al. even observed a protective signal, showing that rescue stenting was associated with a lower risk of sICH even after IVT, compared with patients who underwent EVT alone without achieving recanalization [19]. Alexandre et al. conducted the only study to directly compare patients with and without prior IVT undergoing rescue stenting for ICAS‐LVO, finding no significant increase in sICH or 90‐day mortality [11]. In their cohort, GPIIb/IIIa inhibitor‐based regimens were the predominant intraprocedural strategy (used in nearly 70% of cases); however, the small sample size limited their ability to assess the combined effect of IVT and different antiplatelet regimens.

### 4.3 Safety of Combined IVT and Intraprocedural APT for Acute Intracranial Stenting

IVT with alteplase or tenecteplase promotes systemic fibrinolysis and destabilizes local hemostatic plugs in ischemic brain tissue, an effect that can persist for several hours and leave the vasculature more prone to bleeding once reperfusion is achieved [20]. In parallel, aggressive intravenous APT such as GPIIb/IIIa inhibitors or cangrelor produce an immediate and profound suppression of platelet aggregation, effectively abolishing the capacity to form stable thrombi at sites of endothelial injury or reperfusion‐related microvascular damage [21].

The combined impact of these therapies on hemorrhage risk has not previously been investigated in the context of acute intracranial stenting. Prior work on acute cervical carotid artery stenting for tandem lesions showed that intraprocedural intravenous APT (with GPIIb/IIIa inhibitors or cangrelor) increased the risk of sICH in patients who also received IVT [10]. While extracranial and intracranial stenting differ in several important aspects, these observations provide a useful parallel. Although the pharmacologic rationale suggests the potential for additive or multiplicative effects, our findings indicate that the combined impact of IVT and aggressive APT on hemorrhagic risk is more nuanced. In the setting of acute intracranial stenting for ICAS‐LVO, these pharmacologic effects are compounded by procedural factors: patients often undergo multiple failed thrombectomy passes, balloon angioplasty, and stent deployment across stenotic, fragile vessels [2]. Such maneuvers create additional endothelial trauma, and when combined with abrupt reperfusion, generate a particularly high‐risk environment for hemorrhagic transformation.

Among the four treatment strategies analyzed, the combination of prior IVT with aggressive APT showed a numerically higher crude frequency of sICH‐PH1‐PH2, although this difference did not reach statistical significance (Table 1). However, crude frequencies should not be conflated with the adjusted interaction model. In multivariable analyses incorporating an IVT‐by‐APT interaction term, the interaction estimate was < 1, indicating a less‐than‐multiplicative joint effect (Figure 2). Interpreted conditionally, IVT among patients receiving conservative APT and aggressive APT among patients not receiving IVT were each associated with higher odds of sICH‐PH1‐PH2, whereas the presence of both therapies attenuated the incremental association of the second exposure. Accordingly, patients receiving both IVT and aggressive APT remained at elevated hemorrhagic risk compared with the reference group, but the adjusted data do not support synergistic risk amplification when the two therapies are combined.

Although our study provides real‐world data that may help guide antiplatelet decision‐making, it remains unclear how specific agents interact with IVT and what constitutes the safest and most effective intraprocedural regimen. Larger dedicated studies are needed to establish standardized approaches that balance stent patency against the risk of intracranial hemorrhage. In this regard, forthcoming data from the ongoing Permanent Intracranial STenting for Acute Refractory Large Vessel Occlusions (PISTAR; NCT06071091) [22] and the IntraCranial Atherosclerosis‐Related Large‐Vessel Occlusion Treated With Urgent Stenting trial (ICARUS; NCT06472336) studies will likely provide important insights.

### 4.4 Limitations

This study provides real‐world data on treatment‐related outcomes in patients undergoing intracranial stenting, but several limitations must be acknowledged. First, the retrospective design carries an inherent risk of selection bias and residual confounding, despite adjustments for known clinical variables. Second, the decision to perform intracranial stenting was left to the treating neurointerventionalist, without standardized criteria for its use as a rescue strategy or first‐line approach. Third, there was considerable variability in institutional protocols, as well as in endovascular and pharmacologic strategies across centers. While this variability reflects real‐world practice and enhances external validity, it may also limit the generalizability of our findings as antiplatelet strategies were not standardized across sites (agent, dose, bolus vs. infusion, timing, and duration), introducing residual confounding by indication even after multivariable adjustment. Some continuous covariates were dichotomized in selected analyses to ensure model stability, which may have reduced statistical power. Additionally, site‐level practice variation (procedural workflows, anticoagulation use, imaging cadence, and sICH adjudication processes) may influence outcomes. Importantly, imaging assessment, including grading of hemorrhagic transformation, was performed locally at each participating center without central adjudication, which may have introduced inter‐center variability in outcome classification. Fourth, the registry predominantly includes data from Europe, the USA, and Western Asia, with limited representation from regions where ICAS is more prevalent, which may also restrict generalizability. Fifth, ICAS itself was not uniformly defined across centers and, therefore, could not be included in the analyses. Sixth, subgroup analyses based on clinically relevant criteria, such as rescue versus first‐line stenting, were limited by small sample sizes. Seventh, agent‐level comparisons within aggressive APT (e.g., cangrelor vs. GPIIb/IIIa) were underpowered and unadjusted and should be deemed hypothesis‐generating only. Finally, detailed reasons for withholding IVT in otherwise eligible patients were not captured in the registry and therefore could not be analyzed. The study also did not distinguish between thrombolytic agents (alteplase versus tenecteplase) or analyze outcomes according to timing of IVT or incorporate IVT‐to‐groin puncture (or IVT‐to‐APT) intervals, factors that could plausibly modify hemorrhagic risk.

## 5 Conclusion

Our findings suggest that IVT and aggressive intraprocedural APT each contribute to hemorrhagic risk during acute intracranial stenting when considered in the absence of the other exposure. However, the negative IVT × APT interaction indicates that their joint effect is attenuated on the multiplicative odds scale rather than synergistic. Accordingly, patients receiving both therapies remained at increased risk relative to patients who received none of the treatments. These findings highlight the need for prospective studies to define optimal intraprocedural antiplatelet strategies in IVT‐treated patients requiring emergent intracranial stenting.

## Author Contributions

A.R.‐C. contributed to the conception, drafting the text, acquisition of data, and preparing the figures. L.C.‐C. contributed to drafting the text, acquisition of data, and preparing the figures. E.Ko. contributed to the analysis of data. M.O.‐G., F.D., J.Ka., A.M., S.G., S.Se., A.C., A.Sa., M.Pi., M.M., A.L.‐F., A.E.H., S.Mi., E.Z.‐A., A.A.‐C., M.B., S.Mo., J.A.S., F.G., J.S.‐F., A.A., A.P., J.H., P.M., L.S., E.Ka., J.A., T.M., L.R., F.C., D.R., E.B.‐R., D.S., M.Ab., P.K., T.N.N., C.P.C., A.B., N.A., I.F., D.R.Y., J.H.C., J.R.C., P.V., A.Ö.Ö., Z.U.K., S.Sm., S.A.S., J.Kh., T.G.J., F.B., F.R., P.C., L.A., A.Sid., V.J., P.N., N.N., M.Ps., M.V., J.Z., G.P., Y.A., M.Al., J.F.A., A.Sie., M.Ro., J.P.M., S.Y., M.Ri., A.T., M.Re., and S.O.‐G. contributed to acquisition of data and drafting the text. M.Re. and S.O.‐G. contributed to the conception, design of the study, and drafting the text.

## Funding

The authors have nothing to report.

## Conflicts of Interest

The authors declared the following potential conflicts of interest with respect to the research, authorship, and/or publication of this article: Dr. M.R. reports compensation from Anaconda Biomed for consultant services. Dr. F.D. reports compensation from Medtronic and Balt for consultant services. Dr. M.R. reports compensation from Rapid Pulse for consultant services; stock holdings in Nora; compensation from Sensome for data and safety monitoring services; compensation from Philips for consultant services; stock holdings in Anaconda Biomed; compensation from Vesalio for consultant services; compensation from Cerenovus for consultant services; compensation from Medtronic MiniMed Inc., for consultant services; compensation from AptaTargets for consultant services; compensation from Stryker Corporation for consultant services; and stock holdings in Methinks. Dr. T.N.N. is an Associate Editor of *Stroke*, has served on the advisory board for Brainomix, and is a speaker for Genentech and Kaneka. Dr. J.K. reports Microvention consultancy, financial support from Medtronic, medication supply support from Boehringer‐Ingelheim for the TECNO trial, a research agreement with Siemens Healthineers, and research grants from the Swiss National Science Foundation, Swiss Academy of Medical Sciences and Swiss Heart Foundation (fees paid to institution). Dr. M.M. reports working as consultant for Stryker, Medtronic, Balt, Inspire, iVascular, Accandis; receiving research support from Anaconda, Cerenovus, iVascular, and Stryker; and reports stocks from Basecamp. S.G. reports compensation from Medtronic, MicroVention/Terumo, and Stryker for consultant services. A.S. is a consultant for Cerenovus, Medtronic, MicroVention/Terumo Neuro, Stryker Neurovascular, and has Patents: Patent No. US 11464528 B2. P.N. reports consultant compensations from Balt, Cerenovus, Medtronic, Penumbra, and Stryker. S.O.‐G. is a consultant for Medtronic and Stryker Neurovascular. The other authors report no conflicts.

## Supporting information


**Figure S1:** Study flowchart.
**Figure S2:** Stratified multivariable associations between intraprocedural antiplatelet regimen and sICH‐PH1‐PH2 by IVT status.
**Figure S3:** Univariable associations with sICH‐PH1‐PH2 in patients treated with aggressive APT with prior IVT in prespecified subgroups.
**Table S1:** Intraprocedural antiplatelet used during intracranial stenting.
**Table S2:** Multivariable logistic regression analysis for sICH‐PH1‐PH2.
**Table S3:** Multivariable logistic regression models for secondary outcomes.

## Data Availability

The anonymized data supporting the findings of this study can be obtained upon request from the corresponding author.
