# Supplementary material for: Interaction of Intraprocedural Antiplatelets and Intravenous Thrombolysis in Acute Intracranial Stenting: RESISTANT Registry Subanalysis
Source: Ann Clin Transl Neurol. 2026 Jun 9:10.1002/acn3.70449. Online ahead of print. doi: 10.1002/acn3.70449 (PMC13394748; doi:10.1002/acn3.70449)
Supplement: Supplementary file 1 — Figure S1: Study flowchart. Figure S2: Stratified multivariable associations between intraprocedural antiplatelet regimen and sICH‐PH1‐PH2 by IVT status. Figure S3: Univariable associations with sICH‐PH1‐PH2 in patients treated with aggressive APT with prior IVT in prespecified subgroups. Table S1: Intraprocedural antiplatelet used during intracranial stenting. Table S2: Multivariable logistic regression analysis for sICH‐PH1‐PH2. Table S3: Multivariable logistic regression models for secondary outcomes. [file ACN3-9999-0-s001.docx]

1. **Supplementary** **Material**

**[Supplementary Figure 1](#sf1)**. Study Flowchart.


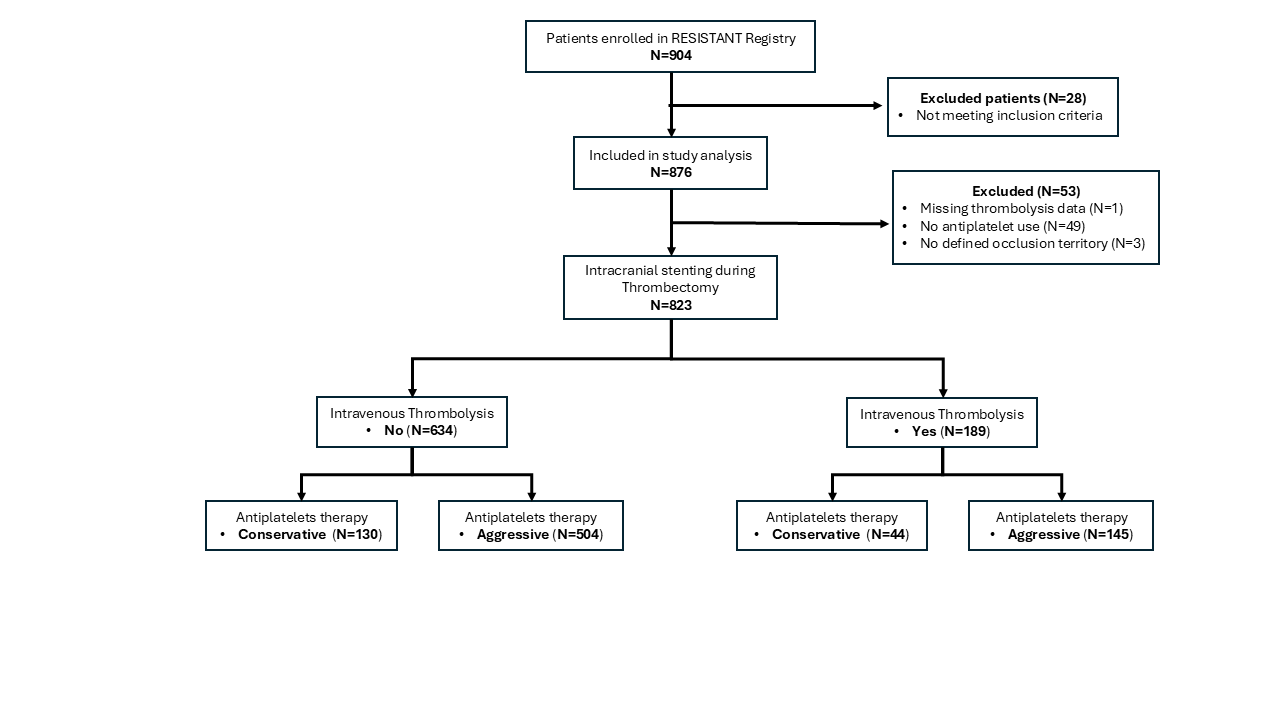


**
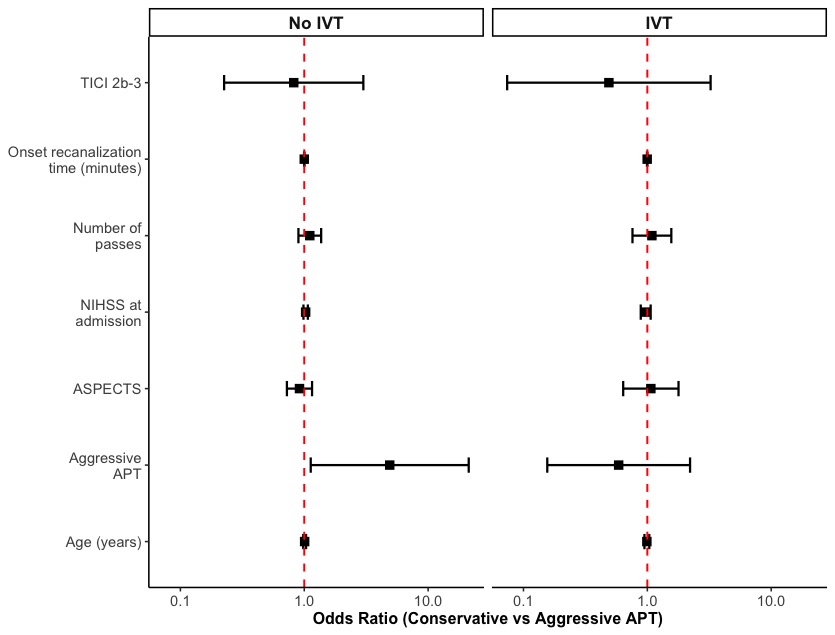
**

**Supplementary Figure 2.** Stratified Multivariable Associations Between Intraprocedural Antiplatelet Regimen and sICH-PH1-PH2 by IVT Status.

**Supplementary Figure 3**. Univariable associations with sICH-PH1-PH2 in patients treated with aggressive APT with prior IVT in prespecified subgroups.


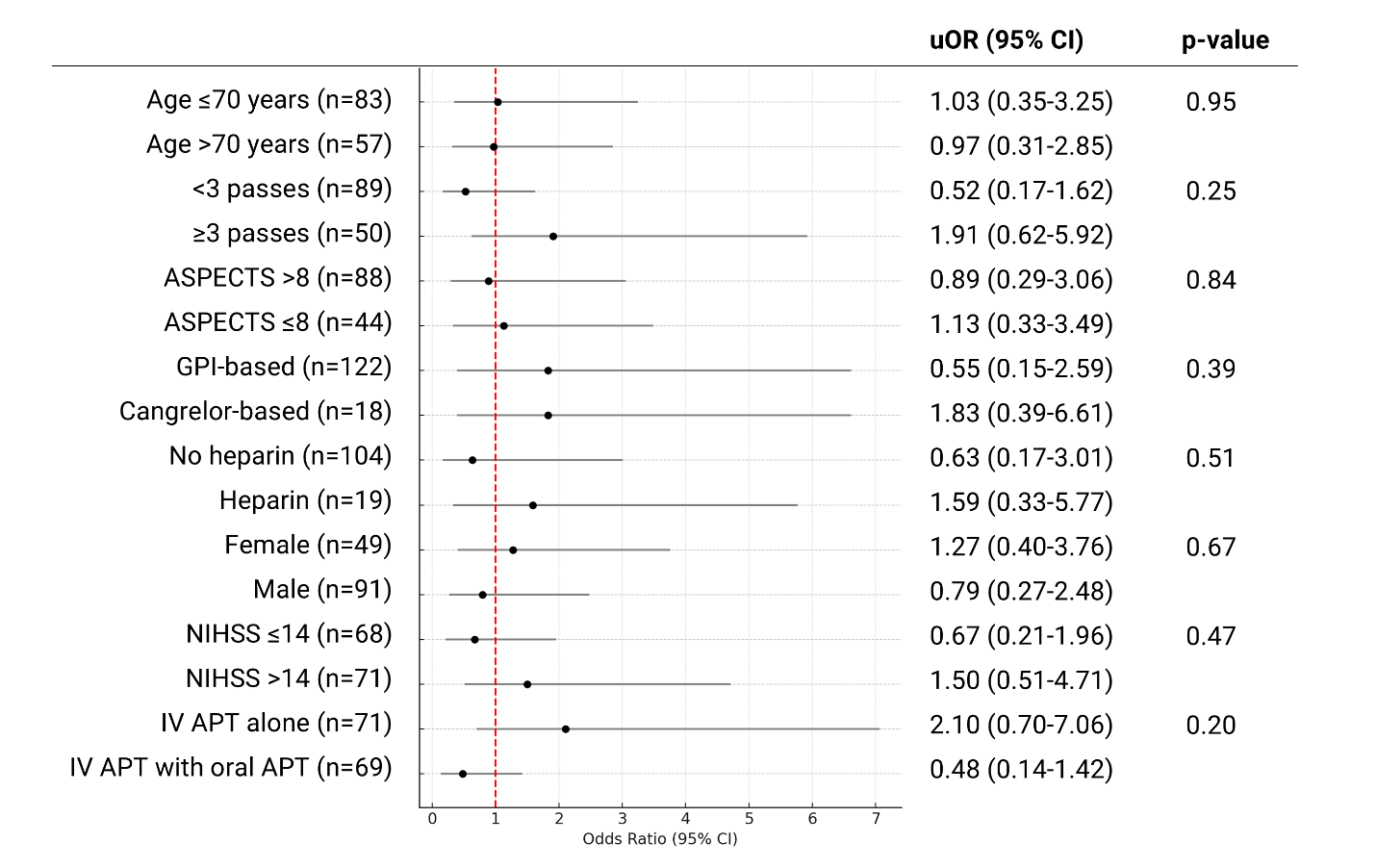


Abbreviations: ASPECTS, Alberta Stroke Program Early CT Score; GPI, Glycoprotein IIb/IIIa inhibitor; NIHSS, National Institutes of Health Stroke Scale; APT, antiplatelet therapy; IVT, intravenous thrombolysis; uOR, unadjusted odds ratio.

| [**Supplementary Table 1**](#st1)**.** Intraprocedural antiplatelet used during intracranial stenting. | | | |
| --- | --- | --- | --- |
| **Group** | **Type** | **Sub-type** | **Number of patients** |
| **Conservative** (n=174, 21.1%) | **SAPT** (n=104, 12.64%) | IV ASA | 93 (53.45%) |
|  |  | Oral ASA | 5 (2.87%) |
|  |  | Oral P2Y12 | 6 (3.45%) |
|  | **DAPT** (n=70, 8.51%) | ASA + Oral P2Y12 | 70 (40.23%) |
| **Aggressive** (n=649, 78.9%) | **Cangrelor** (n=93, 11.30%) | Cangrelor | 75 (11.56%) |
|  |  | Cangrelor + ASA | 15 (2.31%) |
|  |  | Cangrelor + P2Y12 | 3 (0.46%) |
|  | **GPI**  (n=556, 67.56%) | Abciximab | 6 (0.92%) |
|  |  | Abciximab + ASA | 8 (1.23%) |
|  |  | Eptifibatide | 16 (2.47%) |
|  |  | Eptifibatide + ASA | 70 (10.79%) |
|  |  | Eptifibatide + P2Y12 | 12 (1.85%) |
|  |  | Eptifibatide + P2Y12 + ASA | 22 (3.39%) |
|  |  | Eptifibatide + P2Y12 + P2Y12 + ASA | 2 (0.31%) |
|  |  | Tirofiban | 232 (35.75%) |
|  |  | Tirofiban + ASA | 127 (19.57%) |
|  |  | Tirofiban + P2Y12 | 4 (0.62%) |
|  |  | Tirofiban + P2Y12 +ASA | 57 (8.78%) |
| SAPT: Single Antiplatelet Therapy, DAPT: Dual Antiplatelet Therapy, GPI: Glycoprotein IIb/IIIa Inhibitor, ASA: Acetylsalicylic Acid, and IV: intravenous | | | |

| **[Supplementary Table 2](#st2).** Multivariable Logistic Regression Analysis for sICH-PH1-PH2. | | |
| --- | --- | --- |
|  | **OR (95% CI)** | **P-value** |
| Intercept | 0.07 (0.00 – 0.92) | 0.05 |
| No IVT | Ref |  |
| IVT | 1.18 (0.58– 2.27) | 0.64 |
| Conservative APT | Ref |  |
| Aggressive APT | 2.1 (0.92–5.69) | 0.10 |
| Age | 1 (0.98 - 1.02) | 0.96 |
| NIHSS | 1.02 (0.98 - 1.05) | 0.42 |
| Onset-to-recanalization time | 1 (1 - 1) | 0.59 |
| mTICI 0-2a | Ref |  |
| mTICI 2b-3 | 0.75 (0.29 - 2.32) | 0.57 |
| ASPECTS | 0.94 (0.77 - 1.17) | 0.57 |
| Number of passes | 1.1 (0.91 - 1.3) | 0.30 |
| Abbreviations: NIHSS: National Institutes of Health Stroke Scale; mTICI: modified Thrombolysis in Cerebral Infarction; ASPECTS: Alberta Stroke Program Early CT Score. | | |

| **[Supplementary Table 3](#st3).** Multivariable logistic regression models for secondary outcomes. | | |
| --- | --- | --- |
| **Outcome** | **aOR (95% CI)** | ***P* value** |
| **sICH*** |  |  |
| IVT | 1.15 (0.55-2.26) | 0.70 |
| Aggressive APT | 1.95 (0.85-5.29) | 0.15 |
| **Any ICH*** |  |  |
| IVT | 0.94 (0.58-1.49) | 0.78 |
| Aggressive APT | 1.18 (0.72-1.99) | 0.52 |
| **90-day mortality†** |  |  |
| IVT | 0.65 (0.39-1.04) | 0.08 |
| Aggressive APT | 0.81 (0.50-1.32) | 0.39 |
| **Successful reperfusion‡** |  |  |
| IVT | 0.92 (0.48-1.87) | 0.82 |
| Aggressive APT | 2.24 (1.18-4.14) | **0.01** |
| **90-day favorable outcome**** |  |  |
| IVT | 1.03 (0.67-1.57) | 0.90 |
| Aggressive APT | 0.78 (0.50-1.21) | 0.26 |
| **Intraprocedural stent occlusion‡** |  |  |
| IVT | 1.37 (0.77-2.39) | 0.27 |
| Aggressive APT | 0.18 (0.11-0.3) | **<0.01** |
| Abbreviations: APT, antiplatelet therapy; IVT, intravenous thrombolysis; aOR, adjusted odds ratio; sICH, symptomatic intracranial hemorrhage; ICH, intracranial hemorrhage.  *Adjusting for age, NIHSS, onset-to-recanalization time, ASPECTS, reperfusion status, and number of passes.  †Adjusting for age, NIHSS, onset-to-recanalization time, and ASPECTS.  ** Adjusting for age, NIHSS, onset-to-recanalization time, ASPECTS, and reperfusion.  ‡Adjusting for onset-to-recanalization time, and tandem lesion. | | |
